# Metaplastic breast cancer and *BRCA1*: first strong evidence of a link

**DOI:** 10.1038/s41431-023-01441-6

**Published:** 2023-08-17

**Authors:** D. Gareth R Evans, Anthony Howell, J. Howell Sacha

**Affiliations:** 1 Manchester for Genomic Medicine, Manchester University Hospitals NHS Foundation Trust, Manchester, UK; 2 Division of Evolution, Infection and Genomic Sciences, School of Biological Sciences, Faculty of Biology, Medicine and Health, University of Manchester, Manchester Academic Health Science Centre, Manchester, UK; 3 Prevent Breast Cancer Centre, Wythenshawe Hospital Manchester Universities Foundation Trust, Wythenshawe, Manchester, UK; 4 Manchester Breast Centre, The Christie NHS Foundation Trust, Wilmslow Road, Manchester, UK; 5 Division of Cancer Sciences, Faculty of Biology, Medicine and Health, University of Manchester, Manchester Academic Health Science Centre, Manchester, UK

In this issue Corso et al. [1] have carried out the first reasonable sized germline series of testing for *BRCA1* and *BRCA2* in metaplastic breast cancer. Their headline rate of 56% for *BRCA1* is extremely high, but caution is necessary in translating this to all metaplastic cancers as there is likely a strong testing bias.

Metaplastic breast cancers are a heterogenous group of invasive breast cancers which share differentiation toward squamous or mesenchymal-appearing elements [2]. The reported incidence of metaplastic breast cancer can vary from approximately 0.2 to 1.0% of breast cancers depending on their definition [3, 4] and have an incidence rate of 0.6–1.0 per 100,000 women per year in the USA[2] inferring a lifetime risk of less than 1 in 1000. Assessment by intrinsic subtypes of 28 metaplastic breast tumours found the majority were claudin-low or basal-like intrinsic subtypes [5] with most being triple negative [2]. Metaplastic breast cancer has a poorer prognosis compared to other breast cancer pathologies [2, 6] possibly due to greater propensity to haematogenous spread rather than lymphatic, in contrast to Triple Negative Breast Cancer (TNBC) of no special type [2]. Previous reports of treatment have also shown lower response rates to chemotherapy than would be expected for TNBC, perhaps suggesting a lower ‘BRCAness’ phenotype [2]. Up until now there has been no comprehensive assessment of more than a handful of metaplastic breast cancers for germline pathogenic variants in *BRCA1* or *BRCA2*. A previous report of exome sequencing of 30 metaplastic breast cancers with paired normal tissue did not report any mutations in *BRCA1* or *BRCA2* [6], although the genes were not specifically mentioned and it is possible, they excluded germline variants as they were primarily reporting somatic changes [6, 7].

In this edition of the journal Corso et al. [1] assessed 5,226 breast cancer patients who underwent germline genetic testing for the *BRCA1* and *BRCA2* genes, of which 23 (0.4%) were diagnosed with metaplastic breast cancers. They identified 13/23 (56.5%) with a *BRCA1* pathogenic variant compared to 11.5% (597/5,203) with other breast cancer types (*p* < 0.0001). Interestingly one of the *BRCA1* variants was found in one of the two cases with HER2 overexpression which is not usually a feature of *BRCA1* cancers [8, 9]. Although HER2+ breast cancers that are negative for oestrogen and progesterone receptors are more likely to be *BRCA1* driven than those that are oestrogen overexpressed [8]. The preselection based on age and family history for testing in genetics is likely to mean that the rate of *BRCA1* pathogenic variants in metaplastic breast cancer is about 10-fold less based on the detection rates in all breast cancers tested. This is because population testing studies outside clear founder populations only find around a 1% rate for *BRCA1* [10, 11]. For instance, 1.05% in the BRIDGES study [9] compared to the 11.5% in Corso et al [1]. This suggests a rate of nearer 5–6% for metaplastic breast cancers if all had been tested. The 0.4% of breast cancers tested suggest there was no particular bias towards testing metaplastic breast cancers as this is in the middle of the predicted range on a population basis. All the *BRCA1* variant carriers were <53 years of age at diagnosis and 7/13 had a family history of breast or ovarian cancer with 2 being tested for a known variant in the family. Although the detection rate in this study is extremely high this bias in testing should mean a cautious approach to assessing the likelihood of a *BRCA1* pathogenic variant in those with a sporadic metaplastic breast cancer aged >60 years as the detection rates are likely to be very much lower. For instance, the report of exome sequencing came from a cohort of 347 with over 2/3rds of tumours >51years at diagnosis and no *BRCA1/2* variants were reported in 30 metaplastic tumours. Although the age range in the 30 cases undergoing exome sequencing was not recorded [6]. It seems extremely unlikely the authors would have overlooked *BRCA1* variants if they had been seen, but large rearrangements that account for around 20% of *BRCA1* pathogenic changes [12] would not have been detected in this study.

The Corso et al. report [1] clearly has implications for testing and suggest that metaplastic breast cancers should be treated similarly to all other TNBC when selecting for testing. Clearly more data is required on testing those with later onset metaplastic breast cancers. Poly-ADP ribose polymerase inhibitors (PARPi) have demonstrated activity in both adjuvant and metastatic settings in germline *BRCA1* and *BRCA2* Pathogenic Variant carriers and the diagnosis of metaplastic breast cancer should certainly not dissuade clinicians from ordering germline testing if other local criteria are met [2]. The relative absence of variants in *BRCA2* in this study should not deter testing in this gene as they have also been reported and indeed shown to respond to PARPi [12].

Two further pathogenic germline variants were identified in *MLH1* and *TP53*. The *TP53* associated tumour was a spindle cell sarcoma, but interestingly this was also the pathology in two of the *BRCA1* carriers and indeed two *BRCA1* carriers were noted to have squamous type. It is of note that other genes specifically associated with TNBC were not tested in all the samples and in particular more testing of *PALB2, RAD51D, RAD51D*, and *BARD1* [10, 11] would be important in future studies. Overall, this is an important finding that has implications for testing and treatment of metaplastic breast cancer.
